# Characterization of Mineral and Bone Metabolism Biomarkers in a Chinese Consanguineous Twin Family with Primary Hypertrophic Osteoarthropathy

**DOI:** 10.1155/2020/6698878

**Published:** 2020-12-03

**Authors:** Na Li, Yuhang Ma, Yun Jiang, Li You, Yunhong Huang, Yongde Peng, Xiaoying Ding, Li Zhao

**Affiliations:** ^1^Department of Endocrinology and Metabolism, Shanghai General Hospital, Shanghai Jiao Tong University, 100 Haining Road, Shanghai 200080, China; ^2^International Medical Care Center, Shanghai General Hospital, Shanghai Jiao Tong University, 100 Haining Road, Shanghai 200080, China

## Abstract

**Purpose:**

Primary hypertrophic osteoarthropathy (PHO) is a rare, autosomal, recessive genetic disease characterized by digital clubbing, periostosis, and pachydermia. The underlying cause for the pathogenesis of this disease is a defect in prostaglandin E2 (PGE2) degradation, caused by mutations in HPGD or SLCO2A1. In this study, we describe the clinical characteristics, SLCO2A1 mutations, and bone metabolic markers of a PHO pedigree from a Chinese consanguineous twin family.

**Methods:**

Whole blood and urine samples were collected from all the family members. All the exons and exon-intron boundaries of the HPGD and SLCO2A1 genes were amplified using polymerase chain reaction (PCR) and sequenced. The biomarkers of mineral and bone metabolism, including calcium, phosphorus, parathyroid hormone (PTH), 25-hydroxyvitamin D (25(OH)D), bone Gla-protein (BGP), C-terminal telopeptide of type I collagen (*β*-CTX), and urinary calcium/creatinine ratio (Uca/Ucr) were detected.

**Results:**

A homozygous (nonsense) mutation in the *SLCO2A1* gene (c.1807C >T/p.R603^*∗*^) was detected in the proband. Five heterozygous carriers were also identified among his relatives, including his twin brother. The serum BGP (225.5 ng/ml), *β*-CTX (4112 pg/ml), and Uca/Ucr (0.63) levels were significantly elevated, while the 25(OH)D (37.1 nmol/L) level was reduced in the proband. The proband's twin brother displayed increased levels of *β*-CTX (901 pg/ml) and insufficiency of 25(OH)D (67.29 nmol/L), while the other heterozygous carriers only displayed 25(OH)D insufficiency.

**Conclusion:**

The patients with PHO displayed an active state of bone reconstruction. There may be a lack of vitamin D, accompanied by an increase in BGP and *β*-CTX levels. Heterozygous mutations of *SLCO2A1* might lead to mild PHO.

## 1. Introduction

Primary hypertrophic osteoarthropathy (PHO), also known as pachydermoperiostosis, is a rare, genetically and clinically heterogeneous disease associated with defects in the prostaglandin metabolic pathway. The condition is characterized by digital clubbing, periostosis, and pachydermia [1]. Additional symptoms, including arthritis, hyperhidrosis, anemia, diarrhea, gastritis, and hypokalemia, are also reported [2, 3]. In 2008, Uppal et al. first revealed that genetic mutations in the hydroxyprostaglandin dehydrogenase gene (HPGD; MIM 601688), which encodes 15-hydroxyprostaglandin dehydrogenase (15-PGDH), caused autosomal recessive primary hypertrophic osteoarthropathy 1 (PHOAR1; OMIM 259100) [4]. In 2012, Zhang et al. reported that mutations in the solute carrier organic anion transporter family member 2A1 (SLCO2A1, MIM 601460), which encodes prostaglandin transporter protein (PGT), resulted in autosomal recessive primary hypertrophic osteoarthropathy 2 (PHOAR2; OMIM 614441) [5]. Both genes, HPGD and SLCO2A1, are involved in the metabolism of prostaglandin E2 (PGE2). Mutations in these genes lead to failure of PGE2 degradation, resulting in elevated levels of PGE2, which is believed to be the underlying cause for the pathogenesis of PHO [2, 5–8].

PHO has a broad clinical spectrum that affects both skin and bones. It is well known that symptoms in the joints are very common in PHO patients. In a study, 20–40% PHO patients had arthralgia and arthritis, mainly in the knees, ankles, and wrists [9]. Acro-osteolysis of the distal phalanges, periosteal hyperostosis of the long bones, and bone erosions are also commonly observed by radiography [5, 6, 10, 11]. Moreover, bone metabolic markers, such as BGP and *β*-CTX have been found to be higher in PHOAR2 patients than in PHOAR1 patients and healthy subjects [10, 12, 13]. However, to the best of our knowledge, no study has reported the levels of bone metabolic markers in heterozygous carriers from a family with PHO cases. Here, we describe the clinical characteristics, SLCO2A1 mutations, and bone metabolic markers of a PHO pedigree from a Chinese twin family.

## 2. Materials and Methods

### 2.1. Subjects

**Figure 1:**
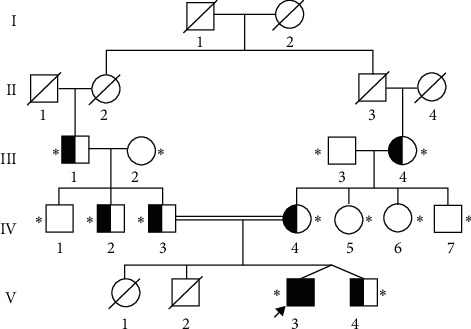
Pedigree of the Chinese family with PHO. PHO is an autosomal recessive genetic disease. The asterisks indicate the members who participated in the study. The arrow indicates the proband. Circles and squares represent females and males, respectively. The filled black symbol represents the affected individual, and the open symbol represents the unaffected individual. The semiblack symbol represents the heterozygous carrier. Double lines represent a consanguineous marriage. Symbol with slash indicates the deceased.

In this study, we included a 21-member Chinese Han family (Figure 1), of which 13 were evaluated both clinically and biochemically. Based on the clinical and laboratory findings, the proband (V-3) was diagnosed with PHO at Shanghai General Hospital, Shanghai Jiao Tong University. The proband and subject V-4 are twins. This study was approved by the Ethics Committee of the Shanghai General Hospital, Shanghai Jiao Tong University. All participants signed informed consent documents before entering the study.

### 2.2. Laboratory Examinations

Fasting blood and morning urine samples were collected from all participants. Mineral and bone metabolism markers, including serum calcium, phosphorus, parathyroid hormone (PTH), 25-hydroxyvitamin D (25(OH)D), bone Gla-protein (BGP), and C-terminal telopeptide of type I collagen (*β*-CTX), were measured using an automated Roche electrochemiluminescence system (Roche Diagnostic Gmbh, Mannheim, Germany) at the central clinical laboratory of Shanghai General Hospital. The urinary levels of calcium were detected, and the values were normalized to creatinine levels (urinary calcium/creatinine ratio; Uca/Ucr) (Siemens Healthcare Diagnostics Inc., Tarrytown, New York, USA) according to the manufacturer's instructions.

### 2.3. Sequence Analysis of the Associated Genes

Informed consent was obtained from the family before blood sampling and DNA analysis. The DNA was extracted from peripheral blood leukocytes using the Puregene kit (Qiagen, Hilden, Germany). The DNA sequences of SLCO2A1 and HPGD were obtained from an online database (GenBank accession No. NC_000003 and NC_000004, respectively). Primers for the two genes were designed using the Primer 5 software. See Table S1 in Supplementary Materials for the detailed sequences. All exons and the exon-intron boundaries of the two genes were amplified using the polymerase chain reaction. PCR was performed in a final volume of 50 *μ*l reaction system containing 200 ng genomic DNA, 20 pmol of each primer, 200 *μ*M dNTPs, 1.5 mM MgCl2, and 2.5 U Taq polymerase (Sangon, Shanghai, China). HPGD-Exon 6 was amplified by an initial denaturation at 98°C for 3 min, followed by 35 cycles of denaturation at 94°C for 30 sec, annealing at 54°C for 30 sec, and elongation at 72°C for 1 min, with a final extension at 72°C for 10 min. All the other exons of HPGD and SLCO2A1 were amplified by an initial denaturation at 98°C for 3 min, followed by 35 cycles of denaturation at 94°C for 30 sec, annealing at 56°C for 30 sec, and elongation at 72°C for 1 min, with a final extension step at 72°C for 10 min. Direct DNA sequencing was performed using an automated sequencer (ABI Prism 3700 DNA Analyzer; Applied Biosystems, Foster City, CA).

## 3. Results

### 3.1. Clinical Manifestations

**Figure 2:**
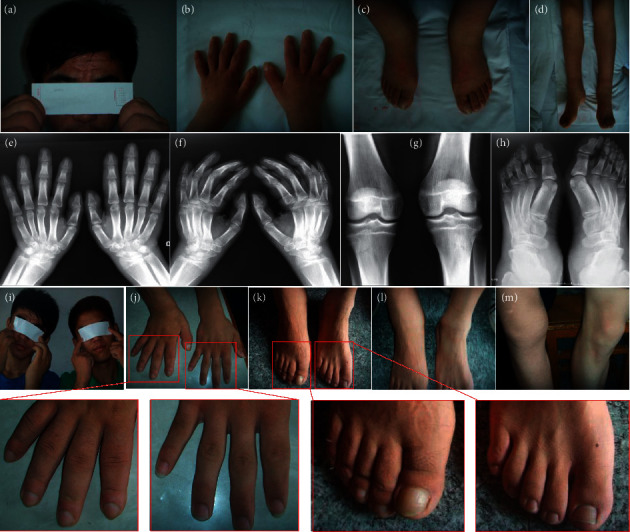
Clinical manifestations of the twin brothers. a–h. Clinical features and radiographs of the proband. i–m. Clinical features of the proband and his twin brother (left, the proband; right, his twin brother). a, i. Thickening and furrowing of the facial skin. b c, j, and k. Digital clubbing of the fingers and toes. d, l, and m. Swelling of the knees and ankles. e–h. Radiographs of the hands, knees, and feet.

The 18-year-old male proband (V-3) was born to unaffected, second-cousin parents. At the age of 16 years, progressive enlargement of the terminal phalanges, facial furrowing, and pachydermia were noticed (Figures 2(a)–2(c)). At 17 years of age, he developed swelling of his ankles and knees (Figures 2(c)–2(d)) and intermittent stomach pain and suffered from severe anemia, for which he received transfusion therapy of red blood cells. He had no arthralgia or watery diarrhea. Radiography revealed swelling of the soft tissue in both hands, knee joints, and feet, symmetrical thickening of the first phalanx and first metatarsal, but no periosteal hyperostosis of the long bones (Figures 2(e)–2(h)). Physical examination revealed no signs of secondary hypertrophic osteoarthropathy, such as heart or lung disease, Graves' disease, or inflammatory bowel disease. Other laboratory analyses, including growth hormone (GH), insulin-like growth factor-1 (IGF-1), and thyroid function were normal. His twin brother (V-4) only had mild digital clubbing (Figure 2(k)). The clinical manifestations of the twin brothers are shown in Figures 2(i)–2(m). PHO is an autosomal recessive genetic disease. His parents and all other relatives were healthy and displayed no signs of PHO.

### 3.2. Genetic Analysis

**Figure 3:**
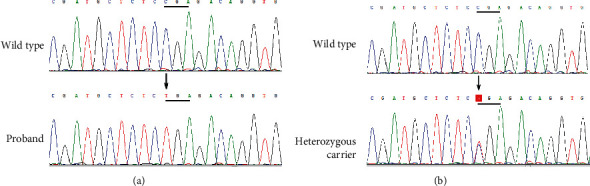
Genetic analysis of the SLCO2A1 gene. (a) The homozygous mutation, c.1807C>T/p.R603^*∗*^, in the proband (V-4). (b) The heterozygous mutation, c.1807C>T in III-1, III-4, IV-2, IV-3, IV-4, and V-4.

The results of the genetic analyses are summarized in Figure 3. Sequence analysis identified the homozygous SLCO2A1 mutation, c.1807C>T in exon 13, which introduced a stop codon at position 603 (p.R603^*∗*^). No mutation was found in the HPGD gene. The proband's grandparents (III-1, III-4), parents (IV-3, IV-4), uncle (IV-2), and twin brother (V-4) were heterozygous carriers of the c.1807C>T mutation (Figure 3).

The c.1807C>T mutation of SLCO2A1 was close to the C-terminus of this gene. The nonsense mutation p.R603^*∗*^ can lead to the truncation of SLCO2A1 and, subsequently, loss of function of the protein.

### 3.3. Biochemical Characteristics of Mineral and Bone Metabolism

The serum and urine levels of biochemical markers for mineral and bone metabolism are listed in Table 1. Laboratory results of the proband (V-3) revealed normal serum calcium, phosphorus, and PTH levels and deficiency of 25(OH)D (37.1 nmol/L). The bone formation marker, BGP (225.5 ng/ml), and bone resorption markers, including serum *β*-CTX (4112 pg/ml) and Uca/Ucr (0.63), were significantly elevated in the proband. The proband's twin brother (V-4) only displayed increased levels of *β*-CTX (901 pg/ml) and insufficiency of 25(OH)D (67.29 nmol/L), while the levels of the other mineral and bone metabolism markers were normal. Among healthy subjects and other heterozygous carriers in the family, most of them had insufficient 25(OH)D levels, while the serum calcium, phosphorus, PTH, BGP, *β*-CTX, and Uca/Ucr levels were almost in the normal range.

## 4. Discussion

Here, we described an affected PHO patient from a Chinese twin family, who displayed the typical manifestations of this disease, including digital clubbing and pachydermia, while his twin brother only displayed mild digital clubbing. All the other subjects in our study did not show any PHO symptoms. Genetic analysis revealed that the proband was homozygous for the mutation c.1807C>T (p.R603^*∗*^) and his twin brother was heterozygous for c.1807C>T in the SLCO2A1 gene. This mutation has been reported in another Chinese PHO patient, who was compound heterozygous for mutations p.R603^*∗*^ and p.G183R [14].

The SLCO2A1 gene, located on chromosome 3q22.1-q22.2, consists of 14 exons, which encodes a 643 amino acid-long, 12 transmembrane-domain organic anion cell-surface transporter, known as prostaglandin transporter (PGT) [3, 14]. PHOAR2, caused by SLCO2A1 mutations, usually develops during puberty, whereas PHOAR1, caused by HPGD mutations, presents symptoms usually in infancy or childhood [2, 10]. A difference is seen not only in the age of onset but also the sex ratio between PHOAR1 and PHOAR2. The male to female ratio is approximately 1 : 1 in PHOAR1, but in case of PHOAR2, mostly males are affected [1, 2, 4, 8, 15]. Therefore, in the current study, since the proband was male and presented symptoms during puberty, PHOAR2 was the first consideration for diagnosis. The results of the genetic analysis supported this diagnosis.

The most frequently observed clinical features in PHO patients are pachydermia, distal clubbing, and periostosis. Pain and swelling in the joints are also common in PHO patients. Zhang et al. found that most of the PHO patients displayed increased serum levels of BGP and *β*-CTX compared to age-matched healthy subjects [10]. In the present study, levels of all the bone formation and resorption markers analyzed were found to be elevated in the proband. SLCO2A1 mutations are associated with aberrant accumulation of prostaglandin E2 (PGE2). It has been reported that PGE2 can promote osteoclast activity and osteoclastogenesis by inhibiting osteoprotegerin secretion and stimulating RANKL expression, as well as production [16]. Treatment of PHO with COX-2 inhibitor, a key enzyme in PGE2 synthesis, has been found to successfully decrease PGE2 and serum levels of BGP and *β*-CTX [10]. Hence, we speculate that the increase in the level of bone resorption markers can be attributed to high serum levels of PGE2, caused by SLCO2A1 mutations. The increase in the level of bone formation markers might be a compensatory increase.

PHO can be caused by the loss of SLCO2A1 function, as a result of either homozygous or compound heterozygous mutations, although only a few patients have been found to carry heterozygous mutations [2, 6, 17]. To date, approximately 100 PHO patients have been reported. However, the bone turnover markers in the heterozygous carriers have not been reported in any study. In the present study, the proband's twin brother was a heterozygous carrier who displayed increased serum levels of *β*-CTX, although the levels were lower than those of the proband. The other heterozygous carriers and healthy subjects had normal bone turnover markers compared to the age-matched healthy subjects [18]. The elevated levels of *β*-CTX in the proband's twin brother may be related to his puberty. However, his *β*-CTX levels were markedly higher than those observed in his peers [19, 20]. Another reason could be that due to the heterozygous mutation, the function of SLCO2A1 is partially impaired, resulting in an increase in the PGE2 level. However, this could not explain the normal *β*-CTX levels observed in other heterozygous carriers. This study has a few limitations. The serum PGE2 levels were not measured, and the functional analysis of the mutation was not conducted.

Notably, the proband had no joint pain, even though he had markedly high levels of bone turnover markers and swelling in his ankles and knees. A possible reason could be that the course of the disease was not adequately long in this patient, and thus, a longer follow-up is required. Different phenotypes may be due to incomplete penetrance, differences in gene expression, or mutation in unknown genes. The molecular and cellular mechanisms of bone metabolism involved in PHO need to be further investigated.

## 5. Conclusions

In conclusion, this study broadened the understanding about the characteristics of biomarkers for mineral and bone metabolism involved in PHO, especially in the heterozygous carriers. Our findings also show that heterozygous mutations in SLCO2A1 might lead to mild PHO.

## Figures and Tables

**Table 1:** Basic information and biomarkers for mineral and bone metabolism of all members in the twin family.

No.	Sex	Age (*y*)	Ca (mmol/L)	P (mmol/L)	PTH (pg/ml)	25(OH)D (nmol/L)	BGP (ng/ml)	*β*-CTX (pg/ml)	Uca/Ucr (mmol/mmol)
III-1	M	64	2.08	1.19	31.80	53.77^∆^	11.67	379.3	0.23
III-2	F	66	2.24	1.28	42.20	72.25^∆^	12.44	566.3	0.47
III-3	M	64	2.12	1.10	22.40	56.85^∆^	13.40	278.1	0.19
III-4	F	63	2.14	1.22	29.40	43.88^‡^	10.44^↓^	469.6	0.27
IV-1	M	42	2.20	1.41	35.60	57.00^∆^	22.52	387.8	0.20
IV-2	M	43	2.09	1.19	32.60	71.67^∆^	14.40	378.1	0.11
IV-3	M	44	2.10	1.33	40.20	57.83^∆^	11.98	281.0	0.15
IV-4	F	44	2.05	1.09	18.20	61.58^∆^	10.99↓	214.4	0.34
IV-5	F	42	2.13	1.32	36.00	58.25^∆^	13.93	209.6	0.12
IV-6	F	41	2.04	1.22	39.80	68.17^∆^	13.54	296.8	0.14
IV-7	M	41	2.20	1.07	20.10	57.42^∆^	13.71	330.4	0.21
^*∗*^V-3	M	18	2.13	1.52	51.70	37.10^‡^	225.50^↑^	4112.0^↑^	0.63^↑^
^#^V-4	M	18	2.14	1.24	39.00	67.29^∆^	37.77	901.0↑	0.23

^*∗*^Proband; ^#^proband's twin brother, ^‡^25(OH)D deficiency; ^∆^25(OH)D insufficiency. Abbreviations: Ca, calcium; P, phosphorus; PTH, parathyroid hormone; 25(OH)D, 25-hydroxyvitamin D; BGP, bone Gla-protein; *β*-CTX, C-terminal telopeptide of type I collagen; Uca/Ucr, urinary calcium/creatinine ratio. Reference range: Ca, 2.03–2.54 mmol/L; P, 0.87–1.45 mmol/L; PTH, 15–65 pg/ml; 25(OH)D, <50 nmol/L, deficiency; 50–75 nmol/L, insufficiency; >75 nmol/L, normal range; BGP, 11–43 ng/ml; *β*-CTX, before menopause, 30–573 pg/ml; postmenopause, 104–1008 pg/ml.

## Data Availability

The data used to support the findings of this study are available from the corresponding author upon request.

## Supplementary Materials

Supplementary Materials Supplemental Table 1: sequences of the primers used for PCR analysis.
