# Supplementary material for: Characterization of Mineral and Bone Metabolism Biomarkers in a Chinese Consanguineous Twin Family with Primary Hypertrophic Osteoarthropathy
Source: Int J Endocrinol. 2020 Dec 3;2020:6698878. doi: 10.1155/2020/6698878 (PMC7732396; doi:10.1155/2020/6698878)
Supplement: Supplementary Materials — Supplemental Table 1: sequences of the primers used for PCR analysis. [file 6698878.f1.docx]

**Supplementary Materials**

**Supplemental Table 1**. Sequences of the primers used for PCR analysis

| Gene | Forward primers (5’→3’) | Reverse primer (5’→3’) |
| --- | --- | --- |
| HPGD-EXON1&2 | CGCTGGCTTGACAGTTTCCT | CCCAGTTGACAGATTGATTCCC |
| HPGD-EXON3 | TGCTTCTAGTCCACAAACCACAC | GGTCATAGTCTGCCAATCCC |
| HPGD-EXON4 | GCAAACCCAAAGAATCCAGG | TTATTGCTTAGGAGTCTCACCA |
| HPGD-EXON5 | CCATTTACTTTCCTTGACTTTC | TATCTCCCCATTACTTATCCTT |
| HPGD-EXON6 | TTACATAATTGTTTCCAGGAGTTG | GCTTGTGTTCATTTCTCAACTGTA |
| HPGD-EXON7 | TACTGATACGATAAAGATGAGAAA | GCTGTTCATTGGGTTTTTGCTT |
| SLCO2A1-EXON1 | CACTCGCTGGCTCAGTCTCC | ACTTCCACTGTCCCATTACTTAG |
| SLCO2A1-EXON2 | CCTGGAGACCCTGGGAAGTAA | GCCTGGGGCTTATCCTCTCTA |
| SLCO2A1-EXON3 | TTGCTTATGCTTGCCCCTTGT | ACCAGAGTTCAGTTGCTCCATAG |
| SLCO2A1-EXON4 | GCCCACATTCCACCTCTCTT | TGTATCCCACAGCCATCCAG |
| SLCO2A1-EXON5 | GCAGGTCTCTTTGGAAGTTGG | GTGAGCGAGTGAGGAGGTGG |
| SLCO2A1-EXON6 | CCCTTCTTCACTACTTCTGCCA | TATTTTCTACCCCCACATCCC |
| SLCO2A1-EXON7&8 | ACAGGTATCGTGGGAGGCAT | TTCTTTCTCCTGGGCAGTCCT |
| SLCO2A1-EXON9 | TTTTTTGTGTAGGCAAGGCA | CTGGGTGACGGAGCGAGACT |
| SLCO2A1-EXON10 | CCCTTTCTGGGTGGTTGGTT | CTGCCTATCCTGGAGCCGA |
| SLCO2A1-EXON11 | GGAACCAGACCCAGAGGACA | TGGACGCTGTGGGACTGAAT |
| SLCO2A1-EXON12 | ATCCAGGGACCACGCTTTG | CTGTGTTCACTTTTCATTTTTCTT |
| SLCO2A1-EXON13 | CCCAGGTCCCTTTTGTCTTCT | CTGTGAGCCCTGAAATGCCT |
| SLCO2A1-EXON14 | CCAGTGGAGGGATGATGAGC | TGGCAAGAAGGGCAGATACC |
